# Dr. Mackay on the Adulteration of the Cantharis Vesicatoria

**Published:** 1842-10-01

**Authors:** John Mackay

**Affiliations:** Edinburgh


*Adulteration of the Cantharis Vesicatoria.* By Mr. John Mackay, Edinburgh.—A species of adulteration of the above insect came lately under my notice, which, from its singularity, I am induced to communicate. I do this more readily, as I am not aware of the circumstances having been mentioned before. From an ounce weight of the flies, I picked, in a few minutes, forty-six various colored glass beads. I estimate the weight of glass in this form to amount to upwards of three ounces in a pound weight of the cantharides. The late Dr. Duncan, in speaking of the adulteration of cantharides, says, " the *melolontha vitis* is sometimes found mixed in considerable numbers with the cantharides. They are easily distinguished by their almost square body ; and as they do not stimulate the skin, should be picked out before the cantharides are powdered." (Duncan's Dispensatory, p. 276.) Dr. Pereira and other writers mention the powder only as liable to adulteration, and chiefly with euphorbium. In Dr. Christison's last work, he says, " in the entire state cantharides are scarcely subject to adulteration in this country. In Germany, the *cetonia aurata* of Fabricius, or *scarabæus auratus* of Linnæus, the golden beetle, is sometimes mixed with it; but this insect may be known by its greater proportional breadth and fat belly." (Christison's Dispensatory, p. 264.) There is, therefore, no evidence of any extraneous matter being introduced to the flies, excepting the golden beetle; which, of course, is at once distinguishable from its appearance.

The addition of glass beads to cantharides, whether it be accidental or fraudulent, ought to be exposed, as the particles of glass, when reduced to powder with the flies, and applied for the purpose of vesication, must prove a source of extreme irritation to the skin.—*Pharm. Journ.*

